# Covering All Bases: A Universal Metazoan UCE Probe Set to Democratize Phylogenomics

**DOI:** 10.1093/gbe/evaf193

**Published:** 2025-10-13

**Authors:** Shahan Derkarabetian, Arianna Lord, Katherine Angier, Julia G Cosgrove, Ella Frigyik, Sara González-Delgado, Breanna Jordan, Paula C Rodríguez-Flores, Shoyo Sato, Lily Shapiro, Gonzalo Giribet

**Affiliations:** Museum of Comparative Zoology & Department of Organismic and Evolutionary Biology, Harvard University, Cambridge, MA 02138, USA; Department of Invertebrate Zoology, San Diego Natural History Museum, San Diego, CA, USA; Museum of Comparative Zoology & Department of Organismic and Evolutionary Biology, Harvard University, Cambridge, MA 02138, USA; Museum of Comparative Zoology & Department of Organismic and Evolutionary Biology, Harvard University, Cambridge, MA 02138, USA; Museum of Comparative Zoology & Department of Organismic and Evolutionary Biology, Harvard University, Cambridge, MA 02138, USA; Museum of Comparative Zoology & Department of Organismic and Evolutionary Biology, Harvard University, Cambridge, MA 02138, USA; Departament de Biologia Evolutiva, Ecologia i Ciències Ambientals, Universitat de Barcelona, Barcelona, Spain; Museum of Comparative Zoology & Department of Organismic and Evolutionary Biology, Harvard University, Cambridge, MA 02138, USA; Museum of Comparative Zoology & Department of Organismic and Evolutionary Biology, Harvard University, Cambridge, MA 02138, USA; Department of Invertebrate Zoology, National Museum of Natural History, Washington, DC, USA; Museum of Comparative Zoology & Department of Organismic and Evolutionary Biology, Harvard University, Cambridge, MA 02138, USA; Marine Biological Section, Department of Biology, University of Copenhagen, Copenhagen 2100, Denmark; Museum of Comparative Zoology & Department of Organismic and Evolutionary Biology, Harvard University, Cambridge, MA 02138, USA; Museum of Comparative Zoology & Department of Organismic and Evolutionary Biology, Harvard University, Cambridge, MA 02138, USA

**Keywords:** animal phylogeny, phylogenomics, systematics, target capture

## Abstract

Biology is in a genomics era, but many researchers may still be alienated from these techniques as a lack of genomic resources remains for many animal groups, necessitating further democratization. Hybrid capture is a popular phylogenomic approach in molecular systematics, with ultraconserved elements being the most popular. However, access to ultraconserved element data is more expensive per sample relative to other commonly used genetic/genomic approaches. Using published genomes, we developed a metazoan ultraconserved element probe set, the universality of which allows multiple research groups working on vastly different animal lineages to share costs and resources across labs. We demonstrated the utility of this probe set both *in silico* against 58 published metazoan genomes and *in vitro* with 130 samples representing 33 metazoan phyla, showing that these probes target loci useful for both deep and shallow level relationships. The proportion of target ultraconserved elements sequenced by the Metazoa probe is equivalent to that of taxon-specific probe sets, but from across all animal phyla. We explored general patterns of ultraconserved element recovery across Metazoa using published genomes and the majority of publicly available ultraconserved element probe sets. The Metazoa probe set is available in three forms: the full set, containing 19,986 probes targeting 2,146 loci, a set containing 10,749 probes targeting 1,022 loci, and the reduced cost set, containing 5,098 probes targeting 466 loci. The development of a universal ultraconserved element probe set should expand the use of genomic data to a much larger segment of the zoological community, with strong potential for broad applications in phylogenomics across all animal phyla.

Significance This study provides new tools to make genomic sampling for phylogenetic studies more accessible to researchers previously unable to conduct genomic studies on their animals of interest. We present a universal ultraconserved element (UCE) probe set that targets all animal phyla and is half the size of a majority of UCE probe sets, thus considerably reducing the cost of genomic-level sampling. The ability to purchase generic, non-taxon-specific probes that can be used by multiple labs at institutions with limited funding will help expand access to the ongoing short-read sequencing revolution.

## Introduction

Since completing the first eukaryotic genome sequencing project in 1996, and with the technological advances of the early 2000s, there is little doubt that biology lives in the era of genomics. The era of genomics is one that goes beyond medical applications thanks to developments that have allowed an increase in sequence read length and accuracy, a decrease in sequencing cost per base, and the ability to work with infinitesimal amounts of DNA. Single-cell genomics (Linnarsson and Teichmann 2016) and telomere-to-telomere chromosome assemblies (Li and Durbin 2024) are now commonly applied to the study of many organisms and these techniques are playing an increasing role in biodiversity sciences. All of these technological and methodological improvements are also significantly benefiting non-model organisms. However, a large part of the zoological community remains alienated from the genomic era due to little access to such techniques and a pervasive lack of resources.

One promising technique to democratize genomics in biodiversity sciences is hybrid capture (or target capture), allowing for the recovery of 100 to 1,000 s of loci with probe sets that can be designed to target specific taxonomic groups. UCEs (Faircloth et al. 2012; McCormack et al. 2012) are the most widely used source of subgenomic scale data because of their utility across multiple taxonomic levels including shallow data sets (e.g. Smith et al. 2014; Derkarabetian et al. 2019a), the relative ease with which probe sets can be designed for specific groups (e.g. Faircloth 2017; Gustafson et al. 2019), and the ability to target samples with degraded or low input DNA (e.g. Blaimer et al. 2016; McCormack et al. 2016; Ruane and Austin 2017; Derkarabetian et al. 2019b). UCE probe sets have been designed for many different animal groups, including tetrapods (Faircloth et al. 2012), marine invertebrates (Quattrini et al. 2018; Geburzi et al. 2024), onychophorans (Sato et al. 2024), arachnids (e.g., Starrett et al. 2017; Kulkarni et al. 2020), and insects (e.g. Faircloth et al. 2015; Faircloth 2017), to mention a few.

UCE probe sets have become increasingly specific in their target taxon through time: for example, in the case of arachnids a probe set was first designed for the entire class (Starrett et al. 2017), then later more specific probe sets were designed to target shallower arachnid orders like spiders and some spider subclades (Kulkarni et al. 2020; Zhang et al. 2023), as well as Acari (Van Dam et al. 2019) and Opiliones (Derkarabetian et al. 2023). This is a useful approach because increasing the specificity of the probe sets tends to increase the amount of loci that can be targeted and sequenced for any given taxon, especially when DNA is highly degraded such as when working with dry or fluid-preserved historical museum specimens (e.g. Derkarabetian et al. 2019b).

While the utility of UCEs is proven, access to UCE data, whether the laboratory work is done in-house or outsourced, continues to be more expensive per sample relative to other methods like Sanger sequencing single loci or RAD-Seq based approaches; while disparity of sizes of metazoan genomes also makes low-coverage whole-genome sequencing (or genome skimming) not ideal for phylogenomic reconstruction using UCEs. The main contributor to these higher costs is the probes themselves. Synthesizing a new probe set for a focal taxonomic group can be prohibitively expensive, particularly at institutions where funding for science is limited, or for labs that are not fully focused on molecular data acquisition. Even when the probes are already available, and thus can be obtained at a lower cost, a lab working on many organisms would need to acquire multiple probe sets. These costs may prevent some research labs from switching to hybrid capture approaches from more traditional approaches, and thus, COI barcoding continues to be the most widespread molecular technique in zoological labs. As such, reducing costs of UCEs for the end user could increase the likelihood of reaching researchers previously limited by funding.

Here, driven by this need of democratizing UCEs, we develop a universal Metazoa UCE probe set that can be used across many different animal taxa and thus shared by multiple research groups. Additionally, we provide two different forms of this probe set, one of which contains a reduced number of probes and therefore costs significantly less than other UCE probe sets. We demonstrate the utility of this universal probe set in multiple ways. Various *in silico* tests were conducted including 58 published genomes spanning nearly all metazoan phyla, as well as shallow level capture in taxa with varying genome sizes. We additionally conduct *in vitro* hybrid capture of 130 metazoan samples spanning 33 extant phyla, testing both deep and shallow phylogenetic divergences within multiple animal groups. Our results underscore the utility of this novel metazoan probe set, demonstrating its strong potential for broad applications in phylogenomics and phylogeography across all animal phyla.

## Results

### Probe Set Design and *in Silico* Testing

Using 58 genomes available in NCBI we designed and tested a universal probe set of UCEs that works across all animal phyla. Following a final coherence check with 58 genomes, our initial probe set contained 19,986 probes targeting 2,146 loci (Metazoa_Final_Full). A reduced probe set was then synthesized (Metazoa_Final_red50), containing 10,749 probes targeting 1,022 loci. The intention of this reduced probe set is to make it accessible to more researchers, but the full probe set is also provided. A subset of this reduced probe set, chosen by selecting the loci present in the 70% occupancy matrix of the reduced cost probe set, contained 5,098 probes targeting 466 loci (Metazoa_Final_red70), which should be even cheaper and should produce enough data for many phylogenetic and phylogeographic studies. We include all probe set files as supplemental files (Supplemental Material); the reduced set (Metazoa_Final_red70) is available from Arbor Biosciences (Ann Arbor, Michigan) as an Expert panel.

RAxML phylogenies of 50% (1,164 loci) and 80% (383 loci) gene occupancy matrices from the initial coherence check with 10 genomes using the Metazoa_Final_Full probe set are available in Fig. S1. Topologies are identical across these two analyses. The IQ-TREE2 phylogeny of the 50% (1,021 loci) gene occupancy matrix for the final coherence check with all 58 genomes using the Metazoa_Final_Full probe set is shown in Fig. S2. The average number of raw loci recovered from across the 58 genomes was 550 (range: 207 to 743) with a mean percentage of targeted loci recovered of 53.9% (range: 20.3% to 72.8%) (Table S1). Generally, despite some deeper level topological differences with recent phylogenomic analyses of metazoans (see Discussion), *in silico* tests indicate that this probe set has phylogenetic utility for deep divergences across Metazoa. Importantly, our intention was not to resolve phylogenetic relationships across Metazoa, but to design a probe set that captures loci from across all Metazoa.

### *In Vitro* Hybrid Capture

Table S4 includes sequencing and UCE processing results. The mean number of raw loci recovered from the Metazoa_Final_red50 set targeting 1,022 loci was 584 (range: 104 to 952), with a mean percentage of targeted loci recovered of 56.94% (range: 10.18% to 93.15%). The average number of loci included in the final 50% occupancy matrix of the Metazoa_Final_red50 was 342 loci (range: 48 to 567). The mean number of raw loci recovered from the Metazoa_Final_red70 probe set targeting 466 loci was 290 loci (range: 44 to 452), with a mean percentage of targeted loci recovered of 62.48% (range: 9.48% to 97.41%). The average number of loci included in the final 50% occupancy matrix of the Metazoa_Final_red70 was 205 loci (range: 29 to 338). The three samples with the lowest recovery proportions included a tunicate sample (*Ciona intestinalis*: MCZ:IZ:166908), one cnidarian sample (*Atolla vanhoeffeni*: MCZ:IZ:150648), and the kinorhynch sample (*Echinoderes truncatus*). In the case of the tunicate and cnidarian, other samples from those orders performed much better, suggesting these were sample-specific issues and not taxon-specific limitations. However, only one kinorhynch was sampled for this study, and genomic resources are limited for these “mud dragons”, so it is difficult to draw conclusions before additional samples are tested.

### Phylogenetic Inference

We generated four 50% occupancy data matrices to illustrate the utility of our probe set in different cases: All Metazoa, Lophophorata, Cnidaria, and Ostreida. The All Metazoa matrix was made up of 130 taxa and contained 597 loci (Table S4). This number of loci in a matrix spanning Metazoa, from both hybridized and genomic samples, illustrates the versatility of the Metazoa probe set across the tree. In the All Metazoa data set, we see many expected phylum-level groupings in our phylogeny, however locus recovery does vary between samples and we see a number of rogue terminal placements (Fig. S3; Table S4). The rogue placement of samples in our tree could be due to a variety of reasons such as variability in number of loci recovered, limited sampling in many areas of the tree, and long branch attraction between distantly related and divergent taxa. We reiterate however that the goal of this study is not to resolve metazoan phylogeny but rather to illustrate the usability of this probeset within phyla across Metazoa.

The final Lophophorata dataset (hybridized samples) contained nine taxa (one outgroup, eight ingroup), and was composed of 351 loci. The final Cnidaria dataset (hybridized samples) contained eight taxa (one outgroup, seven ingroup), and was composed of 303 loci. Ostreida, an order of bivalves that contains oysters, was selected to provide a more densely sampled group on which to test our metazoan UCEs at a shallower level. Our dataset consisted of transcriptomes and genomes publicly available for the group, totaling 33 ingroup taxa spanning seven Ostreida families, plus three Mytilidae (mussel) representatives as outgroup taxa (Table S5). The number of raw loci initially recovered for this dataset ranged from 125 to 892, with an average of 682. Our final dataset 50% occupancy matrix contained 701 loci, with a range of 75 to 663 loci per sample.

### UCEs across Metazoa

**Fig. 1.**
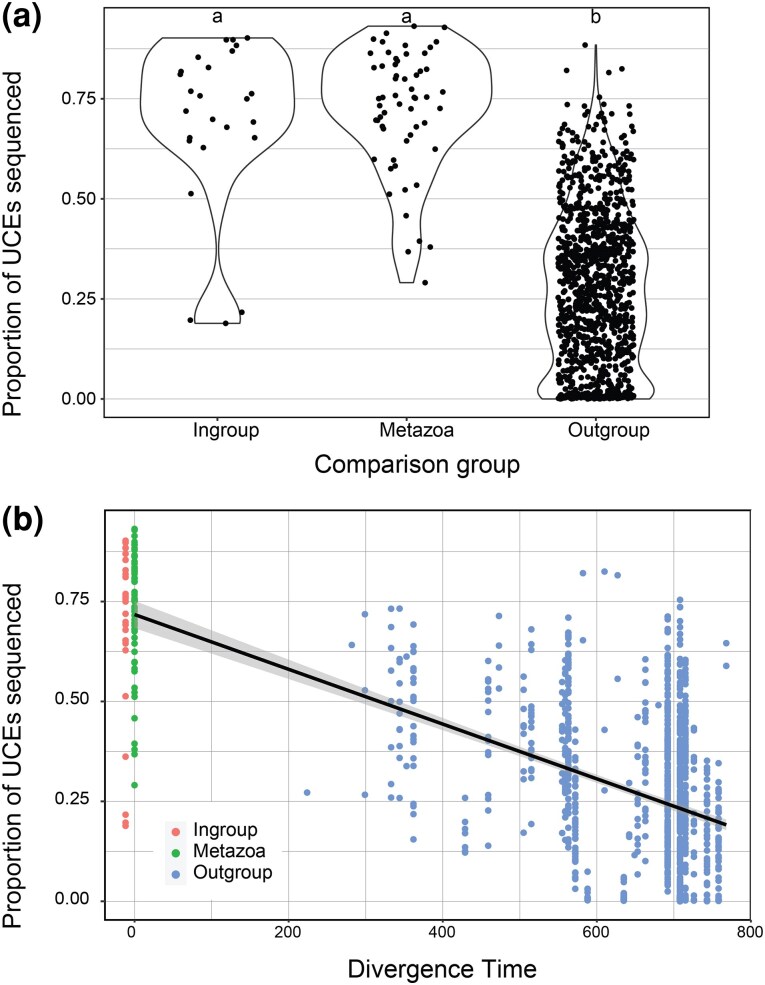
a) Proportion of targeted UCEs sequenced across taxon group comparisons for 21 probe sets across 58 genomes. Comparisons are grouped dependent on whether the genome is the target taxon of the probe set (ingroup), not a target taxon for the probe set (outgroup), or whether the comparison was between any genome and the Metazoa probe set (Metazoa). b) The same comparisons (21 probe sets vs. 58 genomes) plotted against the divergence time of that genome from the probe set in any comparison. Note: the Ingroup points are staggered off the 0 *x*-axis for better visualization of Ingroup and Metazoa points.

**Fig. 2.**
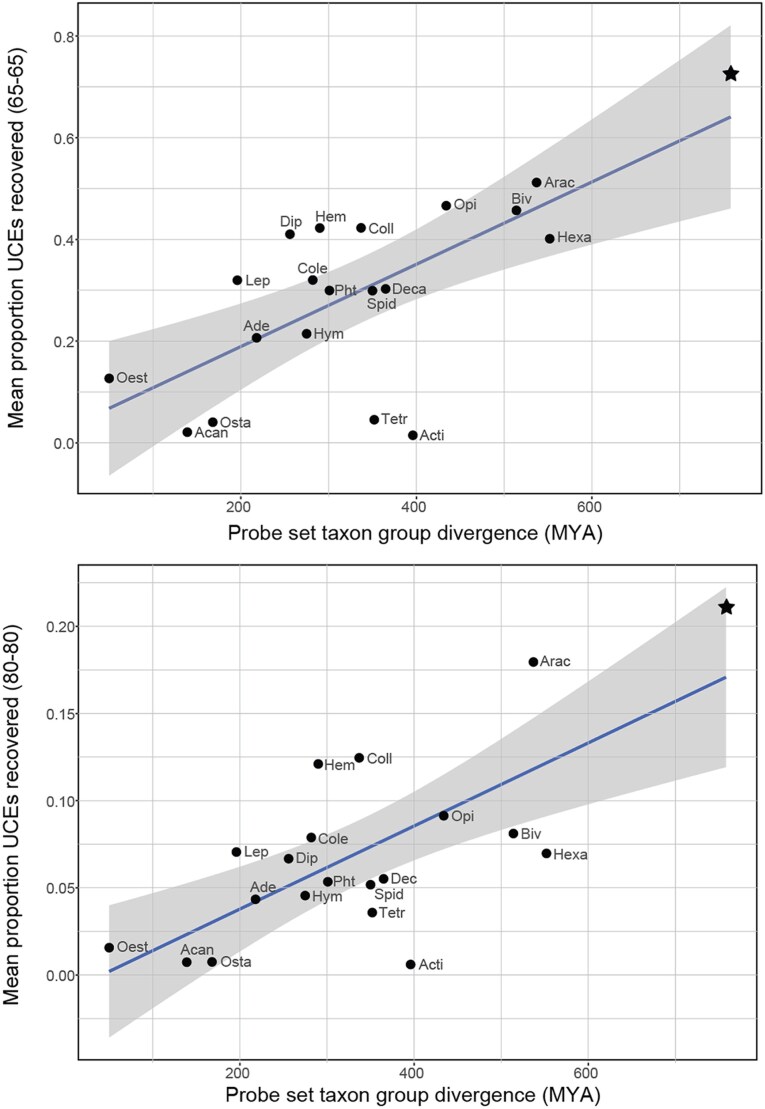
Mean proportion of UCEs recovered for each of 21 probe sets, with each point being an average of pairwise comparison across all 58 genomes used, correlated with the divergence time of the probe set's target taxon. The star represents the Metazoa probe set. Individual probe sets are indicated with abbreviations corresponding to those listed in Table S2. The two plots differ in the min_coverage and min_identity values used: top is 65-65 and bottom is 80-80.

Table S3 shows the results of the pairwise comparisons of matching contigs to probes for the 58 genomes and 21 probe sets for both 65-65 and 80-80 minimum coverage and minimum identity values. Unless stated otherwise, the following results focus on the 65-65 comparisons only. Figure 1a shows the percentage of loci recovered for each pairwise comparison (21 probe sets vs. 58 genomes = 1,218 comparisons) grouped by whether that comparison constituted an ingroup comparison (e.g. an arachnid genome vs. the Arachnida probe set), an outgroup comparison (e.g. an arachnid genome vs. the Tetrapoda probe set), or all comparisons against the Metazoa probe set. The comparisons of the Metazoa probe set to the 58 genomes are equivalent to the ingroup comparisons of taxon-specific probe sets. The mean proportions of loci recovered for each comparison group are as follows: ingroup (mean = 68.6%, range = 18.9% to 90.2%), Metazoa (mean = 72.4%, range = 29.1% to 93.2%), and outgroup (mean = 26.8%, range = 0% to 82.5%). One-way ANOVA indicates significant differences between the mean of comparison groups (*P*-value = <2e-16), and a Tukey multiple pairwise comparison test indicates that ingroup and Metazoa comparison groups are not significantly different (adjusted *P*-value = 0.677), while both are significantly different from the outgroup comparison group (both with adjusted *P*-values = <1e-7). Figure 1b shows the percentage of loci recovered for each of the 1,218 pairwise comparisons versus the estimated divergence time of that comparison, showing a clear negative relationship between age of divergence and UCEs recovered. Figure 2 and Table S2 shows the average percentage of loci recovered across the 58 genomes for each of the 21 probe sets versus the divergence time of the group that probe set targets for both the 65-65 and 80-80 data sets. There is a general increasing trend, where on average a higher proportion of loci are recovered from genomes as the age of the taxon the probe set targets increases. For example, excluding the Metazoa probe set, the Arachnida probe set, which is the second oldest taxon represented at 537 million years of divergence, has the highest average percentage of loci pulled from across all genomes with 51.2% and 17.95% in the 65-65 and 80-80 analysis, respectively.

Genomic characterization of the UCE probe set across exemplar genomes reveals that all three versions (Full, red50, red70) of the metazoan probe set largely target exonic regions. In each instance, on average roughly 79% of UCEs recovered for each genome were exonic, around 18% were intronic, and a very small proportion (around 2%) of UCEs mapped to intergenic regions (Table S6).

## Discussion

### Phylogenomics

In our phylogenetic trees including the 58 genomes (Fig. S2), the placement of three genomes appear questionable: *Oopsacas minuta* (Porifera), *Hydra vulgaris* (Cnidaria), and *Dermacentor silvarum* (Arthropoda). We are uncertain of the cause of these obvious misplacements, but it is unlikely to be the probe set as all other samples from each of those three phyla clustered where expected. Perhaps more importantly, these taxa represent some “extremes” within Metazoa. For example, the sponge *Oopsacas minuta* contains one of the smallest genomes ever sequenced in animals, lacking key metazoan genes (Santini et al. 2023). Additionally, the cnidarian *Hydra vulgaris* genome showed evidence of multiple transposable element expansions, horizontal gene transfer, and simplified gene content and structure that reflect its relatively simple life cycle (Chapman et al. 2010). Finally, *Dermacentor silvarum* is a medically important tick, as *Dermacentor* are external parasites with a variety of mammal hosts.

**Fig. 3.**
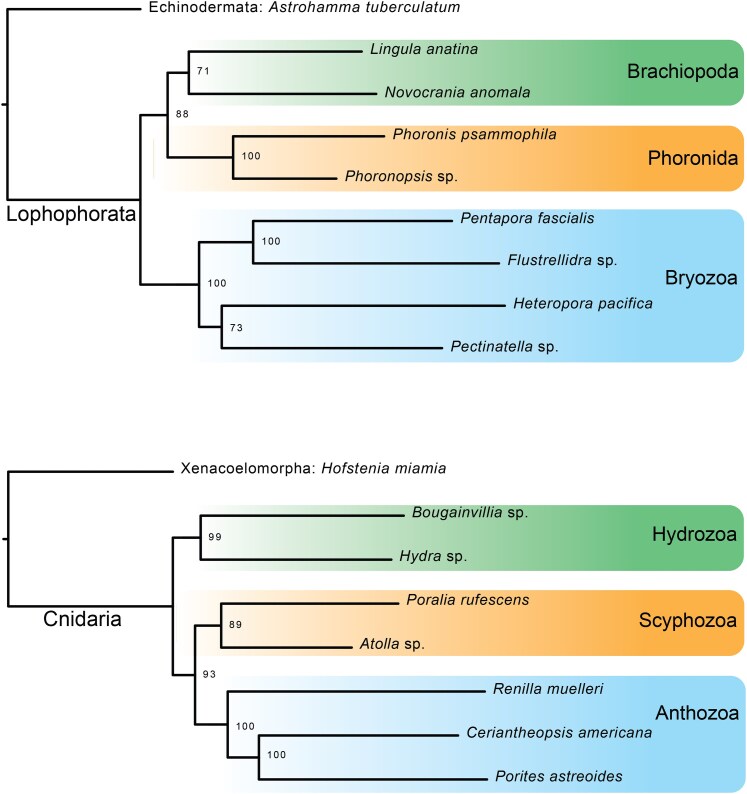
Maximum likelihood phylogenetic results of the *in vitro* tests of the Metazoa_Final_red50 probe set for both Lophophorata (top) and Cnidaria (bottom). Values at nodes are bootstrap support values.

**Fig. 4.**
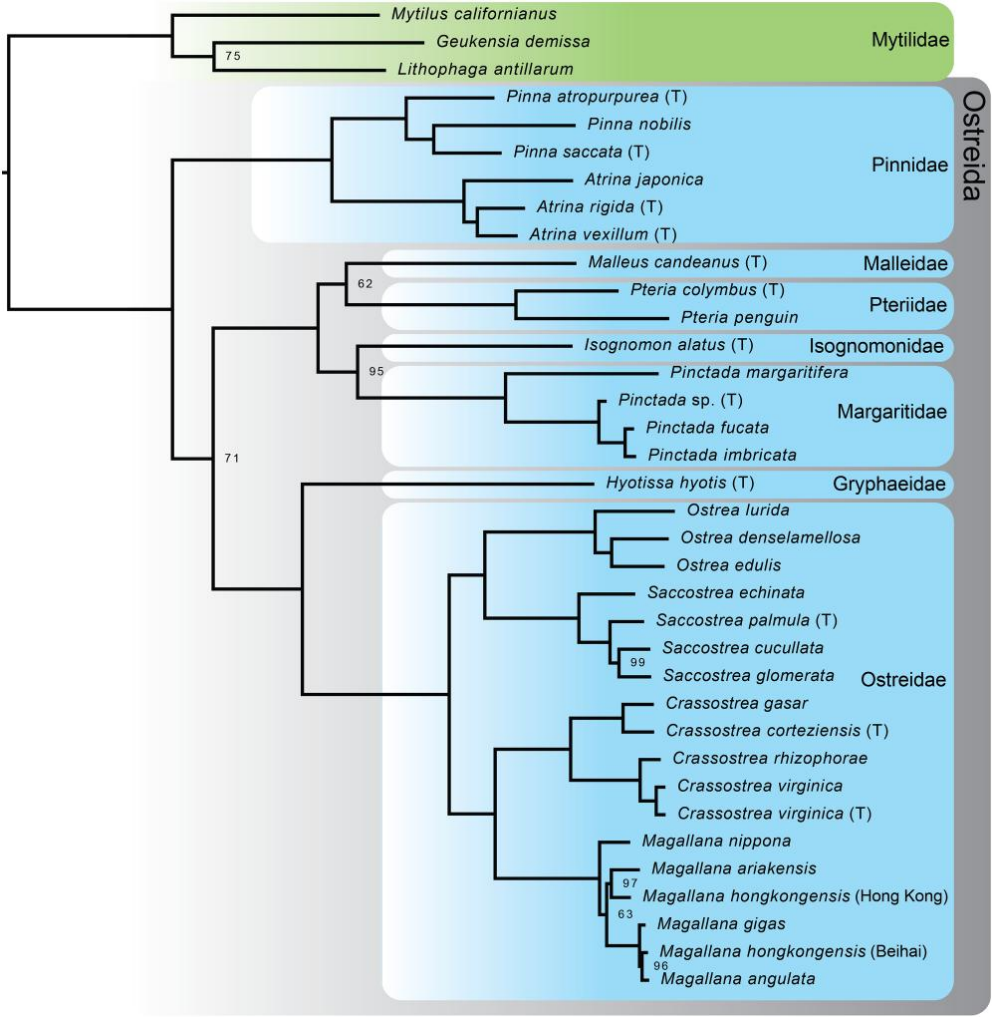
Maximum likelihood phylogenetic results of the *in vitro* tests of the Metazoa_Final_red50 probe set for Ostreida. Values at nodes are bootstrap support values; bootstrap values are 100, unless indicated. Samples with “(T)” are derived from transcriptomes.

The phylogenetic utility of the probe set at shallower levels–as expected–is more robust, as illustrated by our Lophophorata, Cnidaria, and Ostreida datasets (Figs. 3 and 4; Table S5). The relationships in the Lophophorata and Cnidaria trees support generally accepted topologies within these groups (Fig. 3), recovering monophyly of Brachiopoda, Phoronida, and Bryozoa, plus the sister group relationship of Brachiopoda and Phoronida (Hausdorf et al. 2010). The Cnidaria dataset recovered major relationships such as the sister group relationship of Octocorallia and Hexacorallia, and monophyly of Scyphozoa and Hydrozoa. Hydrozoa was recovered as the sister group to all other cnidarians, a result that contradicts current views on cnidarian class-level relationships supporting the monophyly of Medusozoa (Kayal et al. 2018), however this is likely an artifact of this dataset lacking some major cnidarian groups or the ability to resolve Cambrian-level relationships among the cnidarian classes.

At a shallower phylogenetic level, all families and genera represented by more than one taxon were recovered as monophyletic in our phylogeny (Fig. 4). Likewise, we recovered expected relationships of families within Ostreida, including sister group relationship of Gryphaeidae and Ostreidae (Li et al. 2021), a superfamily Pterioidea clade containing Malleidae, Pteriidae, Isognomonidae and Margaritidae, and the family Pinnidae as the sister group to all other Ostreida. This phylogeny represents the densest sampling for a genomic-level phylogeny of Ostreida to date and is compatible with the results from a phylotranscriptomic analysis of Pteriomorphia (Lemer et al. 2016). These results also illustrate that at a shallower level more UCE loci are able to be retained following aligning and trimming steps, generating a robust final dataset. As a potential complementary approach, Metazoa-level universal single copy orthologs (USCOs) were also recently shown to be useful for shallow level species delimitation across Metazoa (Dietz et al. 2024).

### Probe Set Utility and Considerations

In this study, we have demonstrated the universality of our Metazoa probe set through multiple approaches across varying taxonomic levels. In the *in silico* tests, across all taxa, the Metazoa probe set recovered UCEs as well as the ingroup comparisons from existing taxon-specific probe sets (Fig. 1a). This suggests that no matter the metazoan lineage targeted there are “no more outgroups” with respect to the probe set, and all taxa should have more or less an equivalent success rate. With respect to the proportion of targeted loci sequenced, using the Metazoa probe set should provide the same results as if a custom probe set was designed for any given target taxon. Deeper divergence times between the probe set taxon and the target genome lead to reduced efficacy of target capture (Fig. 1b). This is not surprising, as increased divergence time increases the probability of mutations at the probe binding site. Similarly, probe sets designed for taxa represented by deeper divergences recovered a higher proportion of UCEs from non-target taxa (Fig. 2). Recently our probeset was used to bioinformatically extract UCEs from low-coverage genomes of priapulans to help inform phylogenomic relationships within the lineage, further illustrating its utility (Raeker et al. 2025).

There are of course some caveats and considerations regarding a metazoan probe set. We developed a UCE probe set that works across Metazoa, despite the differing nature of “UCEs” in vertebrates compared to that of arthropods. In vertebrates, the UCE regions that end up being selected in probe design tend to be noncoding regions of the genome that are thought to have a role in regulatory functions (Bejerano et al. 2004). Conversely, studies have shown that in arthropods the UCEs that are targeted during probe design are largely coding regions that are exonic in origin (Hedin et al. 2019; Van Dam et al. 2021). More similar to the patterns seen in arthropod probe sets, in our Metazoa probe set, ∼80% of targeted UCEs are exonic regions, with ∼20% targeting intronic regions.

Of course, a universal metazoan probe set has higher potential for capturing UCEs from non-target taxa that more specific probe sets do not necessarily have. For example, UCEs from non-target metazoan parasites (e.g. nematodes, ticks) found within target metazoans may also be recovered using a universal metazoan probe set. There is also potential for the reverse: sequencing non-target host DNA from target parasite taxa, evidenced by nematode and platyhelminth samples being recovered in Craniata (Fig. S3a), both of which are rodent parasites. However, modified bioinformatic processing techniques can be incorporated to decrease the probability of using non-target reads/UCEs in downstream analyses. Available genomes or UCE assemblies of the target taxon could be used as a reference genome, first aligning reads to that genome and only using aligned reads for contig assembly.

This universal Metazoa UCE probe set represents another way of reducing costs associated with genome-scale data collection for phylogenetic analyses. The reduced probe set (Metazoa_Final_red70) with 5,098 probes targeting 466 loci is available as an Expert panel from Arbor BioSciences at significantly reduced costs relative to other Expert probe sets. Costs per sample can also be reduced by incorporating other options. Across most UCE studies, the typical library preparation protocol uses a ½ reaction relative to manufacturer's protocol; this can be reduced even further by doing reactions at ¼ manufacturer's protocol. Similarly, using enzymatic fragmentation library preparation kits eliminates the need (and costs) associated with sonication. Additionally, bioinformatically harvesting UCEs from low-coverage genome sequencing (“genome skimming”) is becoming a hybridization-free alternative to target capture. In this case, the “Metazoa_Final_Full” probe set targeting 2,146 loci is the optimal option.

Genomic data have slowly but steadily permeated into all zoological fields. While decades ago some zoologists may have feared genetic data, today their usefulness for resolving evolutionary questions is no longer in question. Despite this, many zoologists remain left out of the genomic revolution due to a lack of resources. The development of a low-cost *and* universal UCE probe set is aimed to expand the use of genomic data to a much larger segment of the zoological community, specifically to those that had been largely relegated to the use of Sanger sequencing. The low cost of the designed probe set should ensure that many laboratories can obtain this resource. The universality of the probe set should allow multiple research groups to pool resources both for buying the probe set as well as the short-read sequencing costs. The efficacy of the probe set at mid-to-low taxonomic levels (i.e. for groups of species, genera, families) ensures that research questions are not limited to specific taxa for which prior UCE probe sets exist. All these factors should contribute to a new era in systematic zoology where data sets scale up in orders of magnitude while combining results across taxonomic groups.

## Materials and Methods

### Probe Set Design

Probe set design in general followed the standard UCE pipeline of Faircloth (2017) and the associated tutorial “Identifying UCE Loci and Designing Baits To Target Them” available at https://phyluce.readthedocs.io. We used a subset of 10 genomes to design probes (Table S1), selecting the genome of *Aethina tumida* (Arthropoda, Coleoptera; NCBI assembly ID: icAetTumi1.1; NCBI reference sequence: GCA_024364675.1) as the base genome. The *A. tumida* genome was the most complete genome available to us (99.3% BUSCO), in addition to being a chromosome-level assembly, of the most diverse phylum of Metazoa (Arthropoda). Nine other exemplar genomes were chosen to represent the phylogenetic diversity across Metazoa (Table S1). After cleaning, exemplar softmasked genome files were converted to 2bit format, and 100 bp reads were simulated using art (Huang et al. 2012) at ∼2× coverage with an insert size of 200 bp. Simulated reads were then aligned to the base genome using stampy (Lunter and Goodson 2011) and unmapped reads were removed with samtools (Li et al. 2009). Then bedtools (Quinlan and Hall 2010) was used to convert BAM files to bed format, sort bed files, and merge overlapping intervals. We determined locus presence across the genomes (phyluce_probe_get_multi_merge_table), retaining all loci that were shared with the base genome and at least one exemplar genome, following the recommendation of Gustafson et al. (2019) . The fasta sequences were extracted from the base genome (phyluce_probe_get_genome_sequences_from_bed) and a temporary bait set was designed (phyluce_probe_get_tiled_probes) at default settings including 120 bp probes. Duplicates were removed (phyluce_probe_easy_lastz and phyluce_probe_remove_duplicate_hits_from_probes_using_lastz), then this temporary bait set was aligned to the exemplar genomes (phyluce_probe_run_multiple_lastzs_sqlite) with minimum coverage and minimum identity set to 65. Sequences were extracted (phyluce_probe_slice_sequence_from_genomes) and consistent loci were detected (phyluce_probe_get_multi_fasta_table and phyluce_probe_query_multi_fasta_table). We retained all loci that matched the base genome and at least four exemplar genomes; these were used in final bait design (phyluce_probe_get_tiled_probe_from_multiple_inputs) and duplicates were removed.

### *In Silico* Probe Set Testing

This final bait set was then used for two rounds of *in silico* testing, first an initial coherence check against the base genome and all 9 exemplar genomes, then a final check against 58 genomes (Table S1). For both sets of *in silico* testing, probes were first sliced from genomes (phyluce_probe_slice_sequence_from_genomes) with flanking sequences of 400 bp, then aligned to genomes (phyluce_assembly_match_contigs_to_probes) with minimum coverage and minimum identity values of 65. Following matching, sequences were extracted (phyluce_assembly_get_match_counts and phyluce_assembly_get_fastas_from_match_counts), and alignments were created using MAFFT (Katoh and Standley 2013) and Gblocks (Castresana 2000; Talavera and Castresana 2007) with the following settings: −b1 0.5 −b2 0.85 −b3 4 −b4 8. These parameters were selected to retain only the “core UCE” in alignments, based on previous studies (Hedin et al. 2019). For the initial coherence check with 10 genomes, RAxML v. 8 (Stamatakis 2014) was used to build a phylogeny from matrices with varying gene occupancies (50% and 80%, meaning that a locus was selected if present in at least 50% or 80% of taxa, respectively). For the final coherence check with all 58 genomes, IQ-TREE 2 (see http://www.iqtree.org/release/v1.6.12; Nguyen et al. 2015) was used to infer a phylogeny from a concatenated partitioned 50% gene occupancy matrix, using the optimal partitioning strategy found via ModelFinder (MFP + MERGE) (Kalyaanamoorthy et al. 2017), the fast relaxed clustering algorithm (rclusterf), and 1,000 ultrafast bootstrap replicates (Hoang et al. 2018). Phylogenies were examined in TreeViewer v2.2.0 (Bianchini and Sánchez-Baracaldo 2024). Because no metazoan outgroup was included, phylogenies were rooted with Ctenophora based on recent studies (e.g. Schultz et al. 2023). Based on locus and probe count statistics, we designed a final probe set that retained all loci recovered in at least 50% of the 58 genomes (Metazoa_Final_red50). A second reduced probe set included loci recovered in at least 70% of the 58 genomes (Metazoa_Final_red70). The probe set Metazoa_Final_red50 was sent to Daicel Arbor Biosciences (Ann Arbor, MI) for synthesis for our in vitro testing. The Metazoa_Final_red70 is also available from Arbor BioSciences at reduced cost.

### *In Vitro* Hybrid Capture

We tested the Metazoa_Final_red50 probe set experimentally across 130 samples spanning a wide range of metazoan taxa and divergence levels, including nearly all metazoan phyla (Table S4). For all samples DNA was extracted using the DNeasy Blood and Tissue kit (Qiagen Inc.) following manufacturer protocol. Libraries were prepared using the KAPA Hyper Plus kit (Roche) following manufacturer protocol at half reaction volumes with a fragmentation time of 3 min, with the exception of the Onychophora samples, which were 6 min. The number of library preparation amplification cycles was optimized for each specimen (Table S4). Sequence capture followed the standard protocol in the Arbor Biosciences myBaits kit version 5. Reactions were then incubated for 24 h with a touchdown protocol (62 °C for 4 h, 60 °C for 16 h, 55 °C for 4 h). Final hybridized libraries were amplified for 18 cycles with universal Illumina primers and sequenced at 150 bp paired-end (PE) reads on an Illumina NovaSeq X lane at the Bauer Core Facility at Harvard University to at least 2 million reads per sample.

Phyluce v.1.7.2 (Faircloth 2016) was used to process, assemble, and align raw reads into final matrices. Adaptor removal and quality filtering was done with illumiprocessor (Faircloth 2013), a wrapper for Trimmomatic (Bolger et al. 2014). Processed reads were assembled using SPAdes v3.15.4 (Bankevich et al. 2012). Contigs were matched to UCE probes using minimum coverage and minimum identity values of 65 and 65; we used these values because 65-65 has been shown in Arachnida data sets to be an optimal setting to recover the most loci for widely divergent lineages (e.g. Derkarabetian et al. 2019b). Loci were aligned using MAFFT (Katoh and Standley 2013) and trimmed with Gblocks (Castresana 2000; Talavera and Castresana 2007) using the following settings: −b1 0.5 −b2 0.85 −b3 8 −b4 10. Loci were further filtered using CIAlign v1.1.0 (Tumescheit et al. 2022) to remove divergent sequences (<60% identity) and gaps.

### Varied Taxonomic Level Phylogenomics

We tested the performance of our probe set at several taxonomic levels using both *in silico* (published genomes and transcriptomes) and in vitro (newly hybridized samples) approaches. We generated trees from four datasets: All Metazoa, Lophophorata, Cnidaria, and Ostreida. The All Metazoa dataset is made up of all genomes used in probe set design + hybridized test samples (130 samples in total). We also constructed two smaller datasets from the hybridized test samples. The Lophophorata dataset contains 8 ingroup samples and 1 outgroup. The Cnidaria dataset contains 7 ingroup samples and 1 outgroup. All trees were constructed using IQ-TREE2 with 1,500 replicate bootstraps.

The Ostreida dataset was generated by bioinformatically pulling UCEs from available transcriptomes and genomes. This dataset was made up of 33 ingroup samples and 3 outgroup samples. We sourced available genome assemblies and transcriptome SRA reads for members of this group from NCBI. For the transcriptome reads, quality was assessed with fastqc (Chen et al. 2018), and read correction and trimming was done with rcorrecter (Song and Florea 2015) and Trimmomatic-0.39. Transcriptomes were assembled using rnaSPAdes v3.15.4 (Bushmanova et al. 2019). We used Phyluce v.1.7.2 (phyluce_probe_slice_sequence_from_genomes) to slice contigs in the genome assemblies that matched to the Metazoa_Final_red50 probe set. We used the Phyluce command phyluce_assembly_match_contigs_to_probes to match contigs from both the transcriptomes and sliced genome assemblies. We then followed the standard Phyluce pipeline, with additional trimming and alignment performed with CIAlign v1.1.0, and a 50% occupancy matrix was generated.

### UCEs across Metazoa

We calculated the overlap of existing probe sets across Metazoa. This approach is similar to Bossert and Danforth (2018), except we used almost all UCE probe sets published (as opposed to just one) against a more limited set of genomes. To do this, we conducted pairwise matching of UCE probe sets to metazoan genomes. We selected a total of 58 published genomes and matched them to 21 UCE probe sets, including this Metazoa probe set (Tables S1 and S2). These probe sets represent almost all published probe sets available at the time of our analyses. Genomes were selected from as many phyla as possible, sometimes including multiple genomes per phylum. In cases where many genomes were available (e.g. Craniata) we chose the most complete genomes based on BUSCO scores, comparing each genome to the most relevant BUSCO dataset. In addition, we always chose the base genome that was used to create any given UCE probe set that we tested in this study. To conduct pairwise matching of genomes to probe sets, we used Phyluce v.1.7.2, following the “Harvesting UCE loci from genomes” tutorial available at https://phyluce.readthedocs.io. Soft masked genomes were downloaded from their respective NCBI links. Each genome was first converted to 2bit format using faToTwoBit (UCSC Genome Browser; Perez et al. 2025 ). Then for each probe set, the probes were matched to each genome (phyluce_probe_run_multiple_lastzs_sqlite) and extracted (phyluce_probe_slice_sequence_from_genomes) with 300 bp flanking regions on both sides of the core UCE. Following this, for each genome-to-probe set comparison, we matched contigs to probes (phyluce_assembly_match_contigs_to_probes) using two different sets of minimum coverage and minimum identity values (65 to 65 and 80 to 80). UCE loci were then extracted using phyluce_assembly_get_match_counts and phyluce_assembly_get_fastas_from_match_counts. We also included the Metazoa probe set created in this study in these pairwise comparisons (Metazoa_Final_Red50; see below).

Divergence times for the taxon each probe set targets were determined using TimeTree (timetree.org) by specifying that taxon as a group and noting the age of the root node. For certain probe sets (Acanthomorpha, Ostariophysi, Tetrapoda, Adephaga) there was not enough data to compute ages using this approach; in these cases, the age was determined by getting divergence times between two taxa that spanned the root node of that lineage. Divergence dates for the phylum Onychophora versus some taxa could not be acquired through TimeTree; these were acquired from published studies (i.e. Howard et al. 2022). Plots for Fig. 1 were made in R (R Core Team 2024) using ggplot2 (Wickham 2016). Statistical tests (t-tests and ANOVA) were conducted in R using default functions.

For a subset of our exemplar genomes for which necessary files were publicly available we also assessed the genomic characterization of the Metazoa probe set, following Van Dam et al. (2021), to determine the proportion of the UCEs that target exonic, intronic, and intergenic regions of the genome. To do this we utilized the python scripts (https://github.com/calacademy-research/ccgutils/tree/master/uce_types) developed to complement the analyses performed by Van Dam et al. (2021) . We matched probes (across all three Metazoa probe sets) back to their respective genomes using the BLAST-like Alignment Tool (Kent 2002). The resulting m8 files were then filtered to retain only 100% matches over the 120 bp length of the probe. As per Van Dam et al. (2021) a UCE's position within a genome, was identified by taking the UCE's scaffold and/or chromosome and position from the filtered m8 file and searching the base genome's GFF annotation file to identify overlap between each UCE and particular gene features. Summaries of these results were generated using uce_gff_lines.py script and the average percentage of UCEs targeting exonic, intronic, and intergenic regions were calculated across samples for each probe set.

## Supplementary Material

evaf193_Supplementary_Data

## Data Availability

Raw reads for all newly hybridized samples are available on NCBI SRA (Project Accession (PRJNA1268592), accession numbers SRR33745592—SRR33745521. All probe sets, contig assemblies for all newly hybridized samples, R code, and data files are available via Harvard Dataverse (https://doi.org/10.7910/DVN/TAIBRW.).
