# Supplementary material for: Covering All Bases: A Universal Metazoan UCE Probe Set to Democratize Phylogenomics
Source: Genome Biol Evol. 2025 Oct 13;17(11):evaf193. doi: 10.1093/gbe/evaf193 (PMC12571155; doi:10.1093/gbe/evaf193)

**SUPPLEMENTAL FIGURES**

**Supplemental Figure S1.** Maximum likelihood phylogenetic results of the *in silico* coherence tests of the Metazoa_Final_red50 probe set against the base genome and all nine exemplar genomes at two different min_coverage and min_identity values 60-60 (panel A) and 80-80 (panel B). Support values are bootstraps. Values at nodes are bootstrap support values.


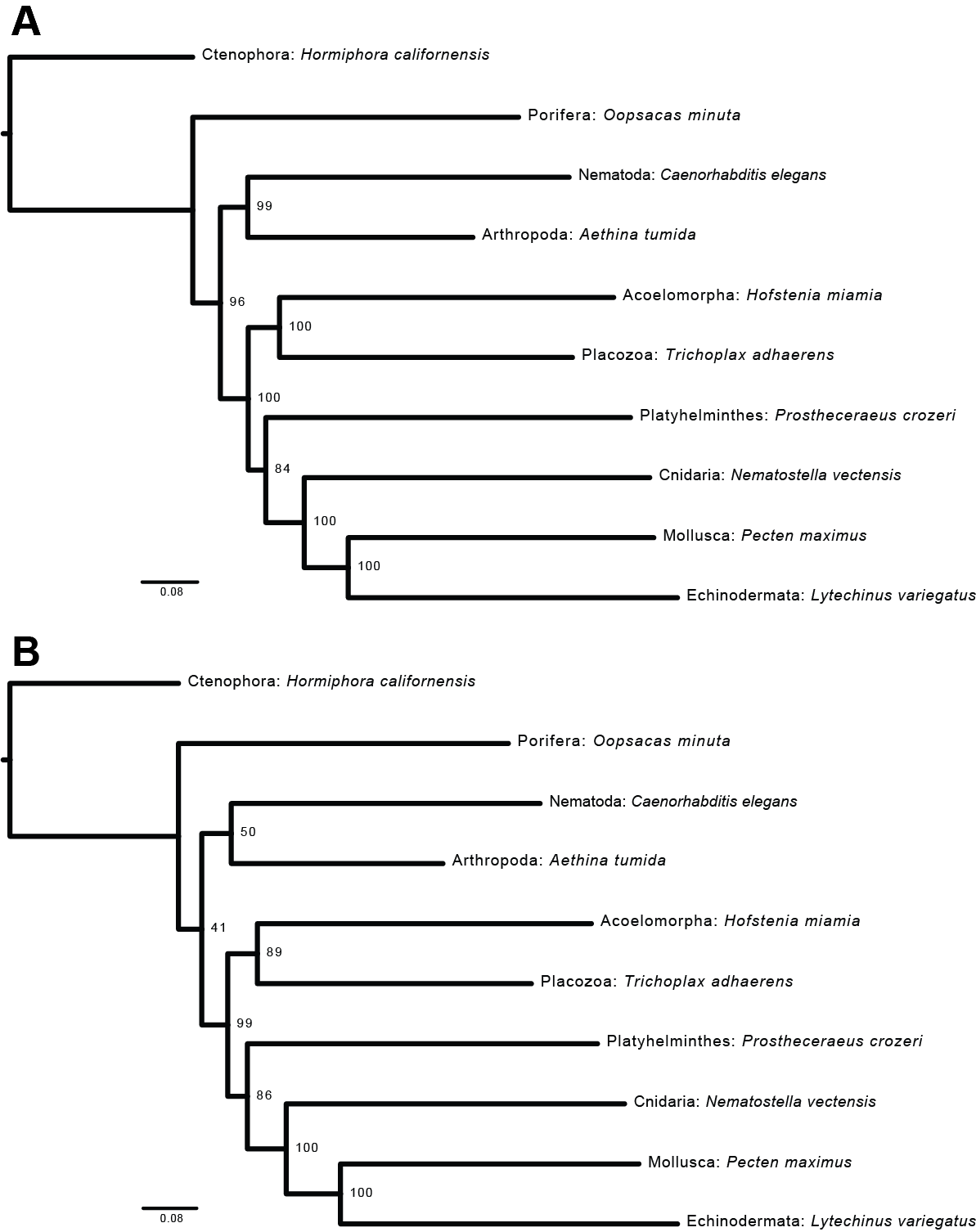


**Supplemental Figure S2.** Maximum likelihood phylogenetic results of the *in silico* coherence tests of the Metazoa_Final_red50 probe set against the 58 published genomes. Values at nodes are bootstrap support values.


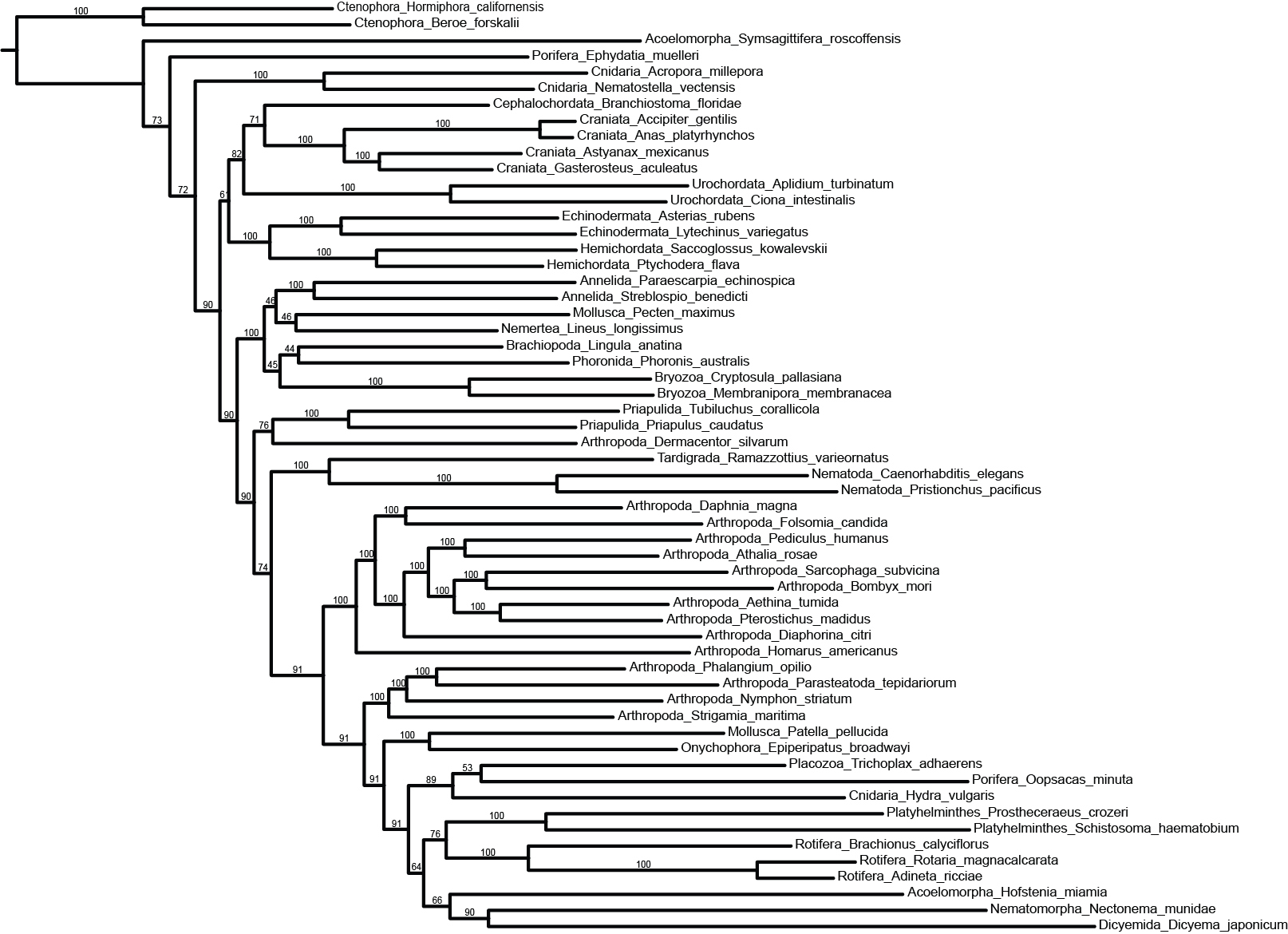


**Supplemental Figure S3A,B (next two pages).** Maximum likelihood phylogenetic results of the in vitro tests of the Metazoa_Final_red50 probe set using 130 samples across Metazoa (All Metazoa data set). Values at nodes are bootstrap support values. Samples represented with red text are samples not recovered in their expected positions.


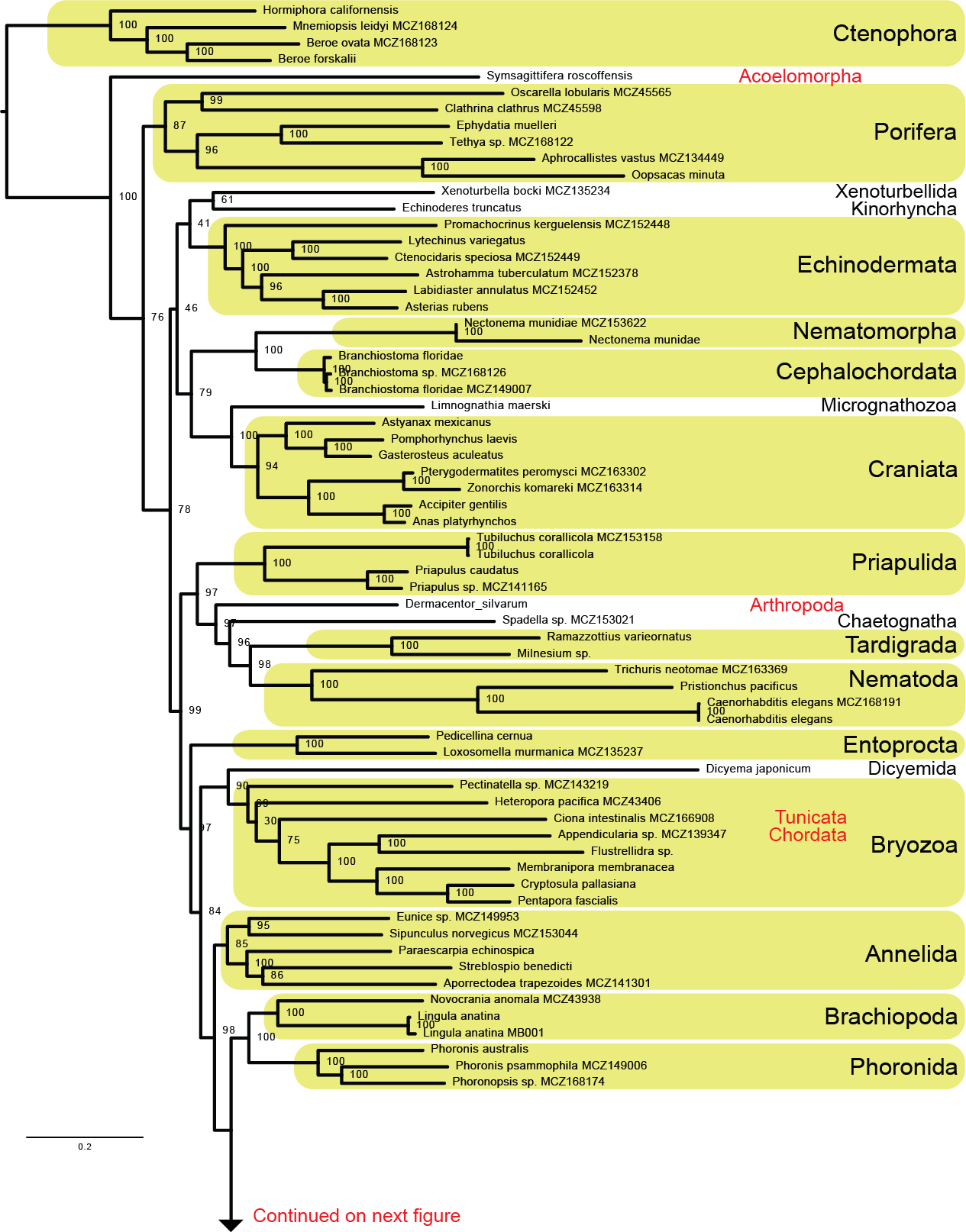


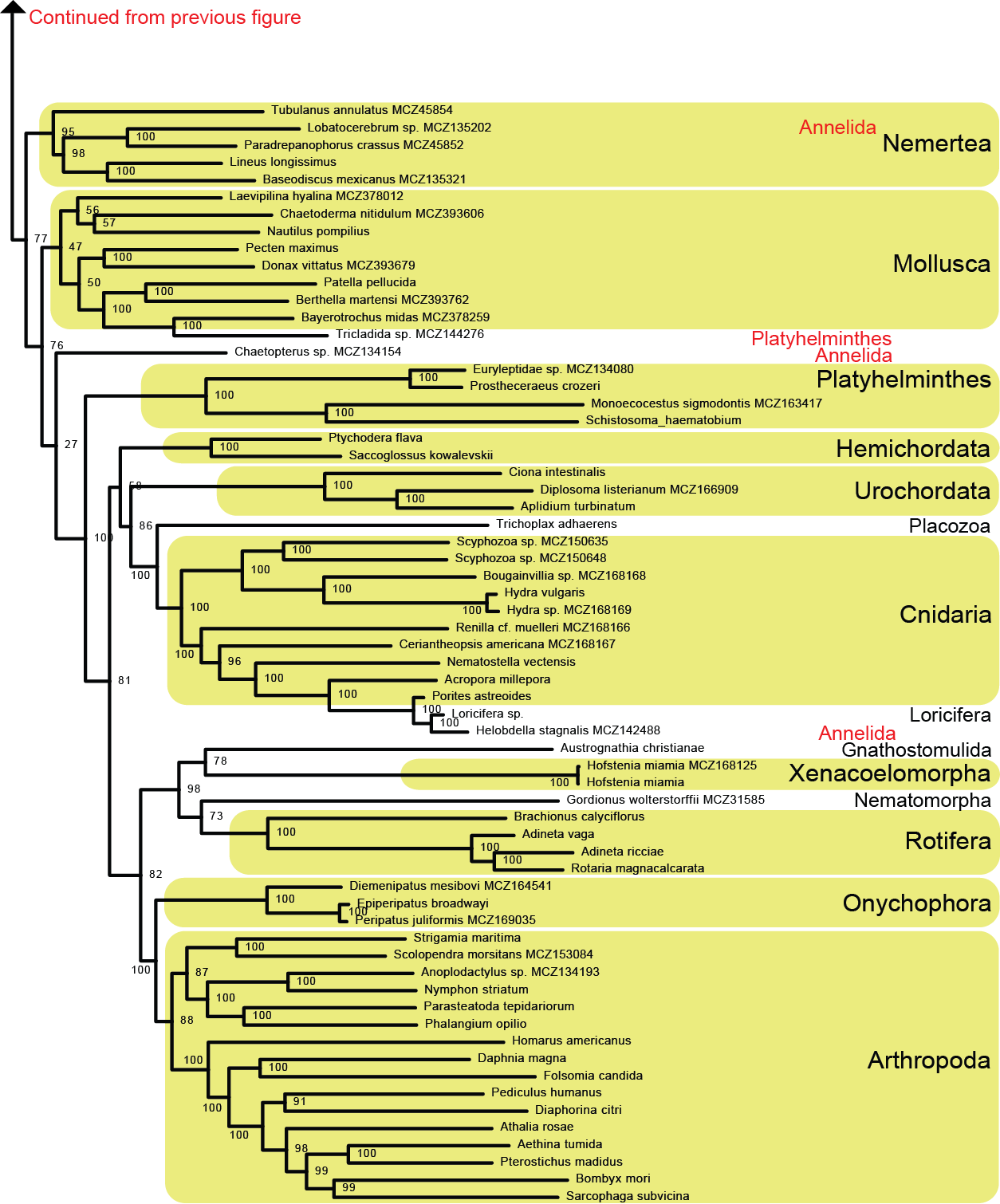

Supplement: evaf193_Supplementary_Data [file evaf193_supplementary_data.zip › SupplementalFigs_v2.docx]
